# SNPs in the interleukin-12 signaling pathway are associated with breast cancer risk in Puerto Rican women

**DOI:** 10.18632/oncotarget.27707

**Published:** 2020-09-15

**Authors:** Angel Núñez-Marrero, Nelly Arroyo, Lenin Godoy, Mohammad Zillur Rahman, Jaime L. Matta, Julie Dutil

**Affiliations:** ^1^Cancer Biology Division, Ponce Research Institute, Ponce Health Sciences University, Ponce, Puerto Rico

**Keywords:** breast cancer, interleukin-12 (IL-12), IL-12 signaling, genetic association, single nucleotide polymorphisms

## Abstract

Interleukin-12 (IL-12) is a proinflammatory cytokine that links innate and adaptive immune responses against tumor cells. Single Nucleotide Polymorphisms (SNPs) in IL-12 genes have been associated with cancer risk. However, limited studies have assessed the role of IL-12 in breast cancer (BC) risk comprehensively, and these were done in European and Asian populations. Here, we evaluated the association of the IL-12 signaling pathway and BC risk in Puerto Rican women. A genetic association study was completed with 461 BC cases and 463 non-BC controls. By logistic regression, IL-12 signaling SNPs were associated with an increased BC risk, including rs2243123 (*IL12A*), rs3761041, rs401502 and rs404733 (*IL12RB1*), rs7849191 (*JAK2*), rs280500 (*TYK2*) and rs4274624 (*STAT4*). Conversely, other SNPs were associated with reduced BC risk including rs438421 (*IL12RB1*), rs6693065 (*IL12RB2*), rs10974947, and rs2274471 (*JAK2*), rs10168266 and rs925847 (*STAT4*), and rs2069718 (*IFNG*). Analyses based in hormone receptors such as estrogen (ER) and progesterone (PR) receptors also revealed protective (for SNPs rs3212227-*IL12B*; rs3024896 and rs3821236-*STAT4*) and predisposing (for rs2069705-*IFNG* SNP) BC associations. Haplotype analysis showed a decreased BC risk for *IL12B* and *STAT4* SNPs, whereas increased risk for *IL12RB1 SNPs*. This study suggests a role of the IL-12 signaling axis and BC risk. SNPs in this pathway may alter IL-12 induced anti-tumor responses and modulate BC predisposition in a population-specific context. Functional studies will be necessary to confirm these findings, which potentially may benefit IL-12 related immunotherapeutic approaches towards BC.

## INTRODUCTION

BC is one of the leading causes of cancer-related deaths among women and constitutes a significant public health burden worldwide [1–4]. BC is highly heterogeneous both pathologically and molecularly with genetics playing an important role for the diverse outcomes this cancer has across populations [5, 6]. The relevant roles of the immune system in BC have been progressively unveiled [7–10]. Therefore, immune systems’ related approaches to BC diagnosis or treatment have gained more attention.

Cytokines are the major cell-signaling proteins of both B and T cells (e. g. interferons, interleukins, chemokines). In addition, cytokines are the key modulators of immune system responses against cancer cells. Many studies have directed their efforts to elucidate the role of these proteins in cancer [11]. Interleukin-12 (IL-12) is an important proinflammatory cytokine for the development of effective anti-tumor immune responses [12, 13]. The main anti-tumor actions of IL-12 are promoted by a specific signaling pathway that includes STAT4 and interferon gamma (IFNG) [14–16]. Despite abundant IL-12 cancer-related research, the roles of IL-12 in the BC carcinogenesis process remains unclear.

Single Nucleotide Polymorphisms (SNP) in the IL-12 genes, *IL12A* and *IL12B*, have been reported to modulate cancer risk across different populations and cancer types [17–20]. Specific SNPs can impair the effectiveness of the IL-12 molecule and the overall anti-tumor capacity of IL-12 related immune cells. However, the main focus of most studies has been the IL-12 molecule itself and have not assessed IL-12 comprehensively, in the context of its main signaling pathway through the STAT4 axis. Minimal published information is available for the relationship of IL-12 SNPs and BC risk. The only reported study which found an association of the rs3212227 SNP on the IL*12B* gene and BC risk was conducted in an European population [21]. This is important given that the relationship between SNPs in immune-related genes and cancer risk varies across populations [22].

Meta-analyses have shown that the cancer risk contribution of IL-12 SNPs varies in the context of the populations’ genetic background [23–25]. Research has tried to address how these differences modulate cancer risk. Yet, the vast majority of these genetic studies have been conducted in populations of predominantly European or Asian genetic background [24]. The Puerto Rican (PUR) population is genetically admixed, with a distinctive combination of European, African and Indigenous American, ancestral contributions [26]. Founder effects have also been reported in Puerto Rico, including in *BRCA2* in the context of the hereditary breast and ovarian cancer syndrome [27]. The interplay of this diverse genetic makeup, in conjunction with the intrinsic biological heterogeneity of BC, has a relevant impact in the immune system’s biology and its role in cancer.

In this study, we assessed the association of the IL-12 signaling SNPs and BC risk in Puerto Rican women for the first time. Our main goal was to test whether SNPs in candidate genes of the IL-12 signaling pathway were associated with BC risk in this unique population. We hypothesized that SNPs in genes pertaining to this signaling pathway were associated with BC risk in PUR women.

## RESULTS

### Description of the study population

The study population consisted of 461 BC cases and 463 non-cancer controls after quality control analysis. The description of demographics, hormonal and pregnancy history risk factors for the study population is summarized on Table 1. The BC cases were older than the controls (*p* < 0.0001), had less education (*p* < 0.0001) and were more likely to be single or widowed than the controls (*p* < 0.0001). Several reproductive BC risk factors were also compared between the two groups. Women with BC were had a smaller number of full-term pregnancies and less children than the controls (*p* < 0.0001). Other reproductive factor such as breastfeeding duration was also significantly different between the cases and the controls, with the controls having longer breast-feeding periods than the cases (*p* = 0.002). These significantly different variables among cases and controls were considered confounders and further adjusted in the logistic regression analyses. The distribution of the genetic ancestry (European, African and Indigenous American admixture) in cases and non-cancer controls is depicted in Supplementary Figure 1. The tumor characteristics for women with BC is presented in Supplementary Table 1.

**Table 1:** Characteristics of the breast cancer cases and non-breast cancer controls

Characteristics	Cases	Controls	*p* value
Age			
*n* (mean ± SD)	463 (51 ± 12.52)	458 (56.23 ± 5.45)	< 0.0001
Civil status			< 0.0001
Single *n* (%)	88 (19.0)	96 (22.0)	
Married *n* (%)	302 (66.0)	233 (54.0)	
Widowed *n* (%)	18 (4.0)	56 (13.0)	
Divorced *n* (%)	52 (11.0)	48 (11.0)	
Educational level			< 0.0001
8th grade or less *n* (%)	8 (1.8)	32 (7.8)	
9–12 grade *n* (%)	132 (28.7)	140 (34.5)	
Associate degree *n* (%)	73 (15.9)	72 (17.7)	
Bachelor degree or above *n* (%)	246 (53.6)	162 (40.0)	
Occupation			< 0.0001
Housewife *n*(%)	109 (23.5)	175 (38.5)	
Works *n* (%)	263 (57.0)	209 (46.0)	
Retired *n* (%)	90 (19.5)	71 (15.5)	
Smoking habits			0.186
Smokers *n* (%)	42 (9.1)	55 (12.1)	
Non-smokers *n*(%)	417 (90.9)	401 (87.9)	
Alcohol Consumption			0.192
Has consumed *n* (%)	82 (17.9)	66 (14.5)	
Never consumed *n*(%)	376 (82.1)	389 (85.5)	
ERT			0.0004
Has used *n* (%)	124 (27.6)	76 (17.5)	
Has never used *n* (%)	325 (72.4)	359 (82.5)	
Number of pregnancies *n*(mean ± SD)	462 (2 ± 1.79)	451 (3 ± 2.53)	< 0.0001
Number of children *n*(mean ± SD)	462 (1.9 ± 1.36)	454 (2.4 ± 1.81)	< 0.0001
Has breastfed			0.372
Yes *n* (%)	248 (53.9)	218 (50.9)	
No *n* (%)	212 (46.1)	212 (49.3)	
Breastfed duration (months)			0.002
*n* (mean ± SD)	422 (9.2 ± 23.57)	403 (18.69 ± 57.03)	

SD: standard deviation; ERT: Estrogen Replacement Therapy.

### Association of the IL-12 signaling SNPs with BC in Puerto Rican women

From 115 SNPs tagging the IL-12 signaling axis, 12 were removed because of call rate < 90% (*n* = 4), poor clustering (*n* = 3), not in Hardy Weinberg equilibrium (*n* = 4) or were monoallelic in the studied population (*n* = 1). Consequently, a total of 103 SNPs remained and were used for further analysis. The list of SNPs tested and relevant information about genomic position is summarized in Supplementary Table 2.

Association between each SNP and BC risk was conducted under four genetic models: allelic, dominant, recessive and additive. A total of 19 SNPs for which the association reached statistical significance (*p* < 0.05) under at least one of the models were further studied (Supplementary Table 3). Also, 3 additional SNPs that were not significant in the overall analysis reached statistical significance after stratifying by estrogen and or progesterone receptor status. All associations were also modeled using the ancestry proportions as covariates (fraction of an individual’s genome from European, African and Indigenous American origin), but did not impact the observed associations (data not shown).

The *IL12A* gene SNP rs2243123 T allele was associated with increased BC risk under the dominant genetic model for the crude (OR 1.35, 95% CI: 1.04–1.75, *P* = 0.02) and for the age and educational level adjusted models (OR 1.34, 95% CI: 1.02–1.77, *P* = 0.04). *IL12RB1* gene resulted with several SNPs associated with BC risk (Supplementary Table 4). SNP rs438421 was significantly associated with BC risk under the allelic model, with A allele having a protective association (OR 0.81, 95% CI: 0.67–0.98, *P* = 0.03). This rs438421 SNP was also associated with decreased BC risk under the dominant model (crude OR 0.81, 95% CI:0.67–0.98; Age, educational level, ERT and breastfeeding duration adjusted model: OR 0.71, 95% CI: 0.48–1.14, *P* = 0.03). Similarly, this rs438421 SNP also resulted in significant risk reduction with BC under the additive model (crude OR 0.81, 95% CI:0.67–0.98, *P* = 0.03; adjusted OR 0.78, 95% CI: 0.54–0.91, *P* = 0.03). For the *IL12RB1* gene SNPs rs3761041 and rs401502 were also associated with increased BC risk under the recessive genetic model (Supplementary Table 4). Lastly for this *IL12RB1* gene, the rs404733 SNP was associated with increased BC risk under the allelic (adjusted OR 1.26, 95% CI: 1.03–1.54, *P* = 0.02) and additive (adjusted OR 1.24, 95% CI: 1.02–1.51, *P* = 0.03).

SNP rs6693065 on the IL-12 receptor gene *IL12RB2* was associated with reduced BC risk under the dominant model (OR 0.74, 95% CI: 0.58–0.96, *P* = 0.03).

*JAK2* gene SNP rs2274471 (OR 0.78, 95% CI: 0.63–0.96, *P* = 0.02), was found to have a protective association under the allelic, dominant, additive and recessive models (Supplementary Table 4). This is a non-receptor tyrosine kinase implicated in signaling by members of the type II cytokine receptor family. From these the most significant associations were for the additive (adjusted OR 0.70, 95% CI:0.54–0.91, *P* = 0.007) and recessive (adjusted OR 0.39, 95% CI: 0.19–0.76, *P* = 0.007) models. Another *JAK2* SNP; rs10974947 was associated with a protective effect under the dominant model (adjusted OR 0.73, 95% CI: 0.55–0.97, *P* = 0.01). In contrast, SNP rs7849191 (*JAK2*) resulted in an increased BC risk under the dominant model (adjusted OR 1.49, 95% CI: 1.05–2.13, *P* = 0.02). The other tyrosine kinase IL-12 signaling gene *TYK2* was also significantly associated with BC risk, with SNP rs280500 increasing BC risk under the allelic (crude OR 1.36, 95% CI: 1.03–1.79, *P* = 0.03), additive (crude OR 1.35, 95% CI: 1.03–1.77, *P* = 0.02) and recessive (crude OR 2.10, 95% CI: 1.06–4.39, *P* = 0.04) models (Table 2). *STAT4* SNPs were also associated with BC in the study population. The rs4274624 in the *STAT4* gene resulted in decreased risk under the allelic (crude OR 0.80, 95% CI: 0.64–0.99, *P* = 0.04), dominant (crude OR 0.74, 95% CI: 0.57–0.96, *P* = 0.02) and the additive (crude OR 0.78, 95% CI: 0.62–0.99, *P* = 0.04) genetic models. SNP rs10168266 (*STAT4*) was associated with reduced BC risk under the crude and adjusted dominant model (Table 2). The rs925847 SNP on the *STAT4* gene had also a protective association under the additive model (crude OR 0.81, 95% CI:0.67–0.99, *P* = 0.04). SNP rs7599504 (*STAT4*) was associated with an increased BC risk under the recessive genetic model (Supplementary Table 4).

**Table 2:** Stratification analysis for ER positive and PR positive/negative tumors associations between selected IL-12 signaling SNPs and BC risk in Puerto Rican women (n = 224)

Gene	SNP	Genetic model	*P* value unstratified crude	Crude OR stratified (95% CI)	*P*value	Adjusted OR stratified^1^ (95% CI)	*P*value	Adjusted OR stratified^2^ (95% CI)	*P* value
*IL12B*	rs3212227	Dominant	0.09	0.69 (0.50–0.73)	**0.02**	0.70 (0.50–0.98)	**0.04**	0.67 (0.46–0.97)	**0.03**
		Additive	0.32	0.74 (0.57–0.96)	**0.02**	0.76 (0.58–0.99)	**0.04**	0.75 (0.56–1.01)	0.06
*IL12RB1*	rs438421	Dominant	0.04	0.66 (0.48–0.92)	**0.01**	0.65 (0.46–0.92)	**0.01**	0.62 (0.42–0.91)	**0.01**
		Additive	0.03	0.76 (0.60–0.96)	**0.02**	0.76 (0.59–0.96)	**0.02**	0.75 (0.57–0.98)	**0.03**
*IL12RB2*	rs6693065	Dominant	0.04	0.66 (0.47–0.91)	**0.01**	0.67 (0.47–0.95)	**0.02**	0.63 (0.43–0.91)	**0.01**
		Additive	0.07	0.54 (0.42–0.68)	0.19	0.84 (0.65–1.08)	0.17	0.74 (0.56–0.98)	**0.04**
*JAK2*	rs10974947	Dominant	0.03	0.67 (0.48–0.93)	**0.02**	0.68 (0.48–0.96)	**0.03**	0.66 (0.45–0.97)	**0.04**
		Additive	0.07	0.73 (0.56–0.96)	**0.03**	0.75 (0.56–1.00)	0.05	0.72 (0.52–0.99)	0.05
	rs2274471	Dominant	0.03	0.65 (0.46–0.90)	**0.01**	0.66 (0.46–0.93)	**0.02**	0.69 (0.47–1.00)	**0.05**
		Additive	0.02	0.68 (0.52–0.90)	**0.007**	0.66 (0.49–0.88)	**0.005**	0.65 (0.47–0.89)	**0.009**
		Recessive	0.16	0.54 (0.25–1.07)	0.09	0.35 (0.13–0.80)	**0.02**	0.25 (0.07–0.65)	**0.01**
*TYK2*	rs280500	Recessive	0.04	2.70 (1.26–5.99)	**0.01**	2.78 (1.19–6.52)	**0.02**	3.00 (1.20–7.62)	**0.02**
*IFNG*	rs2069718	Dominant	0.12	0.73 (0.51–1.04)	0.08	0.65 (0.44–0.94)	**0.02**	0.58 (0.39–0.88)	**0.01**
		Additive	0.04	0.76 (0.60–0.96)	**0.02**	0.70 (0.54–0.89)	**0.004**	0.68 (0.52–0.89)	**0.005**
		Recessive	0.08	0.65 (0.43–0.98)	**0.04**	0.59 90.37–0.90)	**0.02**	0.62 (0.35–0.99)	0.05

OR: Odds ratio; CI: confidence interval; ER: Estrogen receptor; PR: progesterone receptor; neg: negative. ^1^Adjusted for Age and Educational level; ^2^Adjusted for Age, Educational level, ERT and Breastfeeding duration.

Interferon gamma is the main downstream effector of the IL-12 signaling. SNP rs2069718 on this gene was found to be associated with BC risk reduction under the allelic, dominant, additive and recessive genetic models (Supplementary Table 4). From these, the allelic (adjusted OR 0.75, 95% CI: 061–0.91, *P* = 0.004) and additive (adjusted OR 0.74, 95% CI: 0.60–0.91, *P* = 0.004) models resulted in the most significant associations. *TBX21* gene SNP rs2158079 was associated with increased BC risk under the recessive model (Crude OR 2.30, 95% CI: 1.06–3.56, *P* = 0.04). Finally, *PIAS2* SNPs rs10502878 and rs9304337 were associated with reduced BC risk (Supplementary Table 4). The rs2156049 SNP on *PIAS2* gene was associated with increased BC risk. The overall results for the association analysis for all of the 103 SNPs is presented in Supplementary Table 3.

### Stratifying analysis by Estrogen and Progesterone receptor status

BC GWAS studies have uncovered separate susceptibility loci for risk according to tumor characteristics, especially ER and PR status [28–33]. A stratified analysis focusing on the expression of the ER and PR was completed to verify for associations of the IL-12 signaling SNPs and BC risk. From this analysis, new associations emerged. The *IL12B* rs3212227 SNP was found to be associated with a reduced BC risk in a subset of luminal A and B BC tumors (ER positive and PR positive or negative) under the dominant and additive models (Table 2). In addition, a stronger association was observed for SNPs rs438421 (*IL12RB1*), rs6693065 (*IL12RB2*), rs10974947 and rs2274471 (*JAK2*), rs280500 (*TYK2*) and rs2069718 (*IFNG*) after stratifying for luminal tumors (Table 2). Similarly, when assessing the association for the ER negative and PR negative tumors subset, a stronger association was observed for the rs401502 (*IL12RB1*) under the recessive model (Table 3). In addition, SNPs such as 3024896 and rs3821236 (*STAT4*) were associated with a decreased BC risk in the receptor negative tumors (Table 4). Also, the rs2069705 SNP in the *IFNG* gene was significantly associated with an increased BC risk only for the receptor negative tumors subset (Table 3).

**Table 3:** Stratification analysis for ER negative and PR negative associations between selected IL-12 signaling SNPs and BC risk in Puerto Rican women (n = 80)

Gene	SNP	Genetic model	*P* value unstratified	Crude OR stratified (95% CI)	*P* value	Adjusted OR stratified^1^(95% CI)	*P* value	Adjusted OR stratified^2^(95% CI)	*P* value
*IL12RB1*	rs401502	Recessive	**0.009**	2.92 (1.08–7.19)	**0.02**	3.54 (1.28–9.04)	**0.01**	4.14 (1.35–11.82)	**0.009**
*STAT4*	rs3024896	Dominant	0.07	0.58 (0.37–0.99)	0.05	0.58 (0.32–1.00)	0.06	0.47 (0.22–0.92)	**0.04**
		Additive	0.08	0.59 (0.35–0.95)	**0.04**	0.59 (0.34–0.97	0.05	0.46 (0.23–0.86)	**0.02**
	rs3821236	Dominant	0.08	0.59 (0.36–0.95)	**0.03**	0.59 (0.55–0.97)	**0.04**	0.62 (0.34–1.11)	0.11
*IFNG*	rs2069705	Additive	0.30	1.34 (0.95–1.90)	0.09	1.45 (1.01–2.09)	**0.04**	1.48 (0.97–2.26)	0.07
		Recessive	0.29	1.79 (0.97–3.18)	0.05	2.15 (1.15–3.91)	**0.01**	2.35 (1.14–4.66)	**0.02**

OR: Odds ratio; CI: confidence interval; ER: Estrogen receptor; PR: progesterone receptor; neg: negative. ^1^Adjusted for Age and Educational level; ^2^Adjusted for Age, Educational level, ERT and Breastfeeding duration.

Haplotype analysis was also performed for all candidate genes of the IL-12 signaling pathway. The *IL12B* gene a BC risk reduction was evident for the rs3212227G-rs2546892G-rs2569254C-rs3181216A-rs730691T genotypes when compared to the TGCAT genotype (OR 0.62, 95% CI: 0.42–0.91, *P* = 0.01) as seen in Table 4. In addition, the haplotype estimation for specific *STAT4* gene SNPs rs10168266T-rs4274624T-rs925847C genotypes resulted in a protective association when compared to the CCT genotype (OR 0.57, 95% CI:0.39–0.84, *P* = 0.004). On the other hand, the haplotype analysis for the *IL12RB1* SNP rs401502C-rs3761041C-rs404733T-rs438421G showed an increased BC risk when compared to the CCAA genotype (OR 1.36, 95% CI:1.04–1.78, *P* = 0.03). Similarly, the rs401502C-rs3761041T-rs404733A-rs438421G haplotype resulted in an increased BC risk when compared to the CCAA genotype (OR 2.97, 95% CI: 1.04–8.51, *p* = 0.04; Table 4).

**Table 4:** Haplotype association with BC risk of selected IL-12 signaling gene SNPs

Gene							
*IL12B*	rs3212227	rs2546892	rs2569254	rs3181216	rs730691	**Frequency**	**OR (95% CI); *p*value**
1	T	G	C	A	T	0.27	Ref.
2	G	G	C	A	T	0.13	0.62 (0.42–0.91); 0.01
*IL12RB1*	rs401502	rs3761041	rs404733	rs438421		**Frequency**	**OR (95% CI); *p*value**
* 1*	C	C	A	A		0.29	Ref.
2	C	C	T	G		0.25	1.36 (1.04–1.78); 0.03
3	C	T	A	G		0.02	2.97 (1.04–8.51); 0.04
*STAT4*	rs10168266	rs4274624	rs925847			**Frequency**	**OR (95% CI); *p*value**
1	C	C	T			0.49	Ref.
2	T	T	C			0.24	0.57 (0.39–0.84); 0.004

OR: Odds ratio; CI: confidence interval.

## DISCUSSION

IL-12 is a strong proinflammatory cytokine that induces a signaling pathway in which STAT4 and IFNG are key for anti-tumor responses [12, 34, 35]. Immune cells that are closely related to IL-12 signaling, predominantly T cells, can infiltrate breast tumors and induce effective anti-tumor actions [36, 37]. No comprehensive analyses focusing on the role of genetic variation in IL-12 signaling genes in relation to BC risk exists. Furthermore, the limited available studies have been completed within European or Asian populations, which do not fully encompass the genetic diversity that characterizes human populations. In this study we report for the first-time association between SNPs in IL-12 signaling genes and BC risk in Puerto Rican women. We have identified associations of SNPs in several core genes of the IL-12 signaling axis and BC risk in Puerto Rican women, including some that have been reported in other populations, such as *IL12A*, *IL12B*, *IL12RB1*, *TYK2*, *JAK2* and *STAT4*. In the era of precision medicine and immunotherapy, having specific information on how inherited genetics modulates immune responses towards cancer is especially desirable.

Beyond the IL-12 genes, SNPs distributed along the IL-12 signaling pathway resulted significantly associated with BC risk in this study-population. From these, the most notable associations were observed for the SNPs in the IL12 receptor genes, *IL12RB1* (rs401502, rs404733 and rs438421) and *IL12RB2* (rs6693065), the tyrosine kinases genes *TYK2* (rs280500) and *JAK2* (rs2274471), *STAT4* (rs10168266, rs4274624 and rs925847) and *IFNG* (rs2069718). In addition, the SNPs in genes *IL12B* (rs3212227), *STAT4* (rs3024896 and rs3821236) and *IFNG* (rs2069705) were also noticeably associated with BC risk when stratifying by hormone receptor status. Haplotype analysis also contributed to identifying associations in *IL12B, Il12RB1* and *STAT4*.

Previously, IL-12-related genetic associations studies have centered their focus specifically in the heterodimeric IL-12 molecule (encoded by the *IL12A* and *IL12B* genes). Several meta-analyses have summarized the associations of these IL-12 genes and cancer risk [23, 24, 38]. For the *IL12A* gene, the SNPs rs568048 has been one of the most published [20, 38]. This SNP was not found to be associated with BC risk in our study population. However, we found an association of the *IL12A* rs2243123 SNP and BC risk, which to our knowledge is the first report for BC. Ter-Minassian and colleagues reported an association of *IL12A* with neuroendocrine cancer [39]. The *IL12B* gene, which codes for the IL12 p40 subunit has been the most studied IL-12 related-gene in cancer genetic association analyses. A high number of studies have evaluated the relationship of the rs3212227 SNP in *IL12B* with cancer risk. Cervical, hepatic, colorectal, gastric and head and neck cancers are the most common cancers associated with the *IL12B* rs3212227 SNP [23]. The direction of rs3212227 association has been shown to be inconsistent, indicating that SNP it is not likely to be the causative variant underlying this association. There was one report of the association of *IL12B* SNP rs3212227 and BC risk in the Croatian population [21]. Recent meta-analyses identified an increased risk of this rs3212227 with several cancers for populations of European ancestry [23] In contrast, the rs3212227 SNP has been mostly associated with reduced cancer risk, including BC, among populations of Asian ancestry [23]. In our population we only found a significant association of the rs3212227 SNP and BC risk only after stratifying the cases by hormonal receptor status. Causative variants in IL12 may either through altered levels of the protein or structural may result in an attenuated IL-12 signal, given that IL12p40 (*IL12B*) plays the major role in the IL-12 signaling cascade initiation [40, 41]. Because of their central role in the activation of the IL-12 pathway, the *IL12* genes have received the majority of attention. However, a more in-depth information of the contribution of several SNPs in a pathway-context has been proved to be effective to assess cancer predisposition in other studies [42].

The *IL12RB1* and *IL12RB2* genes code for the functional IL-12 receptor which increases its expression upon IL-12 binding, in NK cells, cytotoxic T cells and Th1 helper T cells [15, 43]. For the first time, we report a genetic association of the IL-12 receptor subunits-genes and BC risk in the Puerto Rico population. Previous studies have found associations of *IL12RB1* and *IL12RB2* gene-SNPs with cervical, vulvar and head and neck cancers [25, 44]. In the current study, the association of *IL12RB1* rs401502 and BC was more pronounced in hormonal receptor negative tumors. In addition, rs438421 was significantly associated with lower BC risk under all genetic models assessed. Currently, the molecular mechanisms underlying these associations remain unknown, and it cannot be excluded that the observed associations are the results of other variants located in close proximity. It is possible that certain variants increase risk through a loss-of-function effect, resulting in reduced IL-12 signal transduction in related immune cells. In contrast, protective variants may trigger an enhanced IL-12R activity thereby promoting IFNG production predominantly in cytotoxic T cells. We also report the association of *IL12RB2* rs6693065 with reduced BC risk in the Puerto Rico population. The IL12 beta 2 subunit of the IL-12R, plays a central role in the signaling pathway leading to the development of anti-tumor Th1 T cells [45, 46]. SNPs in the *IL12RB2* gene associated with BC reduction may enhance IL-12 signaling pathway in cytotoxic T cells and promote Th1 cell differentiation, both of which promote anti-tumor actions at tumor sites.

Further downstream in the IL-12 signaling pathway, the Janus kinases TYK2 and JAK2 target STAT4 for further activation. Here we found that *TYK2* rs280500 was significantly associated with increased BC risk, independently of BC subtype. This SNP has previously been found to be associated with colorectal cancer [47], and the risk for several autoimmune diseases such as colitis [48]. Other studies have linked the contribution of variants in *TYK2* with cancers, including BC [49, 50]. Interestingly, the role of TYK2 is not restricted to the IL-12 signaling cascade as this gene is known to crosstalk with other immune and cellular proliferation pathways [51]. Therefore, this association may result from functional variants in *TYK2* that either promote a reduced function and consequently a less effective signaling of anti-tumor cytokines such as IL12, or increase the tyrosine kinase activation of molecules that are related to proliferation and sustained proinflammatory responses. The JAK2 kinase is also involved in other cytokines signaling besides the IL-12. SNPs in the *JAK2* gene have been associated with cancer risk, such as cervical, gastric, and prostate cancers [52–54]. In this study, the rs2274471 SNP in *JAK2* was also associated with BC risk in Puerto Rican women. Slattery *et al*., 2013, reported a protective association of rs2274471 with colon-rectal cancer risk [47]. Although this SNP is novel for BC risk, other studies have also found associations between rs2274471 and cancer risks, including prostate and colorectal cancers [47, 55]. *JAK2* SNPs related to protective association with BC may promote a more efficient signaling pathway and the eventual activation of STAT4. This in turn may enhance IFNG secretion and the anti-tumor actions that this molecule promotes.

STAT4 is the main IL-12 message-transducer molecule of the IL-12 pathway, and *STAT4* gene variants have been implicated with an increased risk of several cancers, including stomach, colon, lung and breast [50, 56]. This gene has also been associated with autoimmune and inflammatory diseases such as lupus erythematous, rheumatoid arthritis, psoriasis, colitis among others [57, 58]. In this study we found a significant association of the rs4274624 *STAT4* SNP and BC risk. This is the first published study finding a protective BC risk association for this SNP. Previously, this SNP was reported to be associated with higher hepatitis B-related cancer risk [59]. We also report a significant association of the *IFNG* gene SNP rs2069718 and BC in our cohort, which has been previously associated with autoimmune disease risk [60]. Specific SNPs in *STAT4* and *IFNG* could be key for the promotion of the IL-12 pathway anti-tumor actions in NK, macrophages and T cells.

While this study presents the first comprehensive analysis on the role of inherited variants in the genes of the IL-12 signaling axis and BC risk, it does present some limitations. First, the cases and non-cancer controls have not been matched and came from different clinical settings. While such factors can be accounted for in the regression analysis, this method is not as robust as working from a matched set. For instance, we observed that the associations of rs404733 in the *IL12RB1* gene SNP was only significant after correcting for education level, which is used as a measure of socio-economic status. There was an imbalance in the education levels between controls and cases, which may be a consequence of recruitment sites. Controls, are identified in family medicine and internal medicine clinics, where patients with chronic diseases would be followed up. The IL12 cytokine family have been implicated in cardiovascular diseases including atherosclerosis, stroke and myocardial infarction [61]. Type II diabetes progression has also been linked to dysregulation of the IL12/STAT4 axis [62]. It is therefore possible that the association between genetic variants in *IL12RB1* and breast cancer be confounded by education level as a result of an increased prevalence of chronic diseases in the control group, due to recruitment strategies. Second, given that there was not strict correction for multiple comparisons, the limited samples size of our cohort and magnitude of *P* values observed, our result will need to be validated in other cohorts of similar ancestral composition or further functional work will be needed to validate these associations.

In conclusion, this study supports a role for the IL-12 axis in the genetic predisposition of BC. It is possible that inherited variations that result in modulation of the IL-12 pathway, either through structural or regulatory changes participate in shaping the anti-tumor immune responses that are promoted by this cytokine. Functional genetic studies will be required to elucidate the mechanism through which these variants impact BC risk. Understanding the genetic basis to cancer risk, including in understudied populations, is a key element in identifying individuals or populations at higher cancer risk that require increased surveillance. This work may also contribute to promote IL-12-related targeted immunotherapeutic approaches.

## MATERIALS AND METHODS

### Ethics statement

The methodology of this study was conducted following the principles included in the Helsinki Declaration.

### Study population

The Ponce Health Sciences University Institutional Review Board approved this study (IRB # 070918-JD and #130207-JM). Each participant signed an Informed Consent form, providing permission to collect a blood sample and to review their pathology reports. All participants completed a 7-page epidemiological questionnaire requesting demographic variables. Participants in this study were selected from a BC cohort recruited from 2006 to 2012 as described in Matta et al. 2012 [63]. Out of these, the final study-population consisted of 924 Puerto Rican women (461 cases and 463 controls). All participants were 21 years or older. Cases were recruited through Oncologic hospitals and private physician offices (oncologists and surgeons) and were recently diagnosed (prior to initiation of any form of therapy such as chemotherapy, radiotherapy, surgery) with a histopathologically confirmed breast carcinoma. Only cases with primary and metastatic breast carcinoma tumors, rather than secondary to other type of cancer, are included. The pathology report from each patient is obtained to know the tumor grade, tumor size, presence of axillary’s lymph node metastasis, and other clinically relevant information. The cases were also screened for *BRCA1*/*BRCA2* mutations in order to exclude those who were *BRCA1/2-*positive. The non-cancer Controls were recruited from individuals visiting gynecological and primary care medical offices for their routine mammography. All controls had a negative mammography within the last 6 months and a clinical breast examination during that period. Any potential participants (cases or non-cancer controls) with known autoimmune conditions was excluded from the current study. All the qualified participants completed a questionnaire including demographics information, hormonal and pregnancy history as well as life-style related questions. The participants also provided a venous blood sample for DNA extraction.

### Tag SNP selection and genotyping

The tag SNPs in the IL-12 signaling pathway genes of interest were selected by a bioinformatics approach. We used the University of California Santa Cruz Genome Browser (UCSC) to obtain the candidate-gene position [64]. Information on the IL-12 signaling candidate-gene SNPs for the Puerto Rican-population was obtained from the 1000 Genomes Browser [65]. The tag SNPs were selected using Haploview [66]. Tag SNPs were selected with the following criteria: (1) SNPs located in one of the eleven genes comprising the IL-12 signaling (Supplementary Table 4); (2) each SNP had a MAF ≥ 0.1 in the Puerto Rican population; (3) each SNP had a r2 ≥ 0.8. A total of 115 tag SNPs in the eleven IL-12 signaling were selected for genotyping. High quality extracted DNA was only included for the genotyping assay. Multiplexed SNP Mass EXTENDED assay was designed using Sequenom MassArray Assay Design 3.0 Software. Sequenom MassARRAY RS1000 was used for genotyping [67]. A set of 106 single nucleotide polymorphisms (SNPs) that can discriminate indigenous American, African, and European ancestry was used to estimate the proportion of genetic ancestry in cases and non-cancer controls was genotype on the same platform. This panel has been described previously [68].

### Genotyping analysis quality control

SNPs-tests with call rates lower than 90% were excluded. All final analyzed tSNPs were in Hardy Weinberg Equilibrium (HWE). Individual samples with genotyping calling rates lower than 70% were excluded from the final analysis. Concordance of duplicated samples was assessed with Plink and these were also eliminated from the final analysis [69]. Global ancestry proportions were estimated from AIMs using ADMIXTURE under a supervised model at k = 3. Reference populations consisted of 42 Europeans (Coriell’s North American Caucasian panel), 37 West Africans (non-admixed Africans living in London, United Kingdom, and South Carolina), and 30 indigenous Americans (15 Mayans and 15 Nahuas) [70].

### Statistical analysis

The genotype-phenotype association was evaluated using the dominant, allelic, additive and recessive genetic models [71]. Frequency distributions differences were calculated by Pearson’s chi-square test. R version 3.2.1 implemented in R Studio was used for the statistical analyses [72]. Haplotype analysis was done for the dominant model using SNPStats software [73]. Associations between the candidate-gene SNPs and BC risk were estimated by obtaining the odds ratio (OR) and the 95% confidence interval by logistic regression. In the analysis we corrected for BC confounders. A *p*-value < 0.05 was considered statistically significant based on a two-tailed test.

## SUPPLEMENTARY MATERIALS







## Abbreviations

BC: Breast cancer
SNP: Single Nucleotide Polymorphism
IFNG: Interferon gamma
PUR: Puerto Rican
OR: Odds ratio
CI: Confidence interval
HEW: Hardy-Weiberg equilibrium
