# Supplementary material for: SNPs in the interleukin-12 signaling pathway are associated with breast cancer risk in Puerto Rican women
Source: Oncotarget. 2020 Sep 15;11(37):3420–31. doi: 10.18632/oncotarget.27707 (PMC7500104; doi:10.18632/oncotarget.27707)
Supplement: Supplementary file 1 [file oncotarget-11-3420-s001.pdf]

SNPs in the interleukin-12 signaling pathway are associated with breast cancer risk in Puerto Rican women

SUPPLEMENTARY MATERIALS

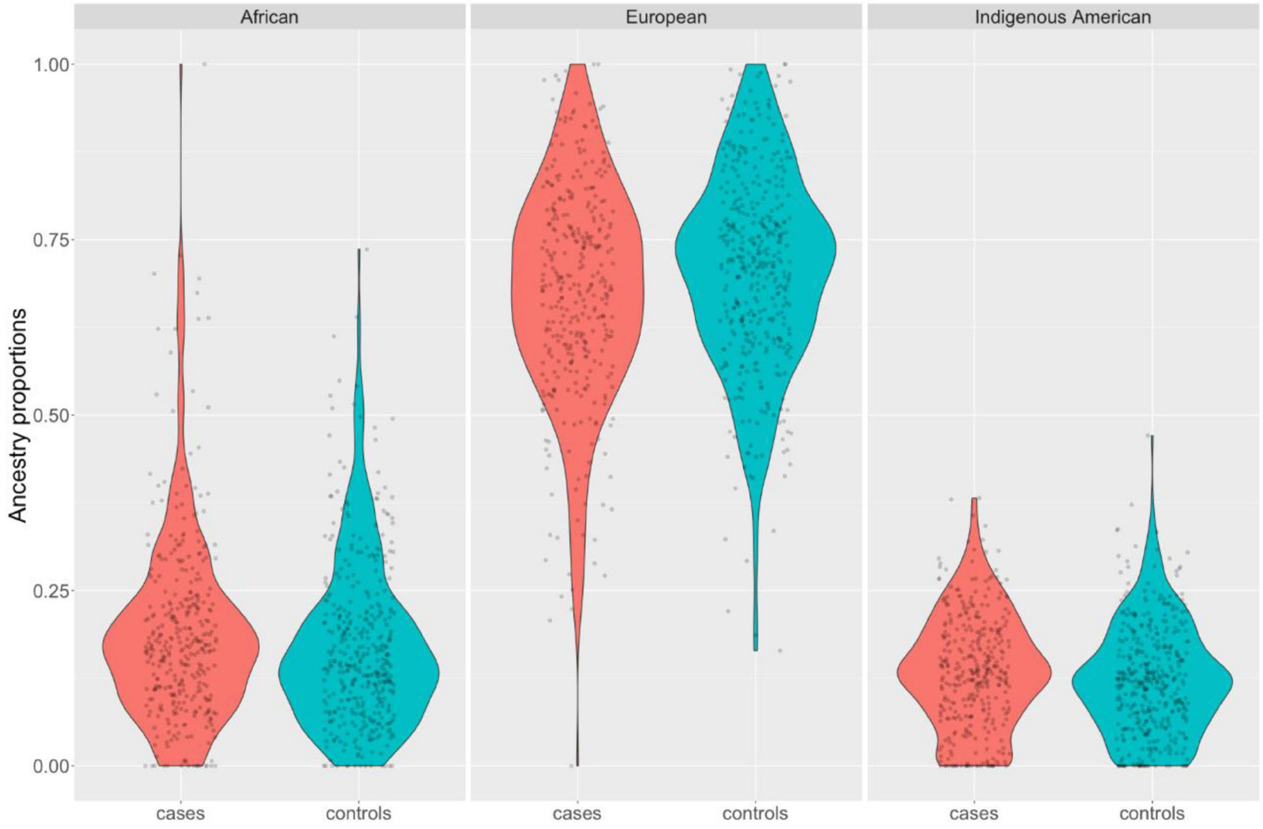

Supplementary Figure 1: Distribution of the genetic ancestry (European, African and Indigenous American admixture) in the study samples. The distribution of the genetic ancestry from study cases (pink) and controls (green) is depicted.

Supplementary Table 1: Tumor characteristics of the study-population

Characteristic	Frequency
Type of carcinoma	*n* (%)
Ductal	393 (90.3)
Lobular	30 (6.8)
Ductal + Lobular	9 (2.9)
Tumor invasiveness	*n* (%)
In situ	89 (20.6)
Invasive	343 (79.4)
Tumor grade	*n* (%)
I	66 (17.0)
II	197 (50.6)
II	126 (32.4)
Tumor size	*n* (%)
Less than 2 cm	153 (63.2)
More than 2 cm	89 (36.8)
Lymph node invasiveness	*n* (%)
Yes	68 (37.8)
No	112 (62.2)
Receptor status	*n* (%)
Estrogen Positive (ER pos)	266 (75.7)
Estrogen Negative (ER neg)	85 (24.3)
Progesterone Positive (PR pos)	230 (66.3)
Progesterone Negative (PR neg)	117 (33.7)
	***n* (%)**
ER pos PR pos/neg	229 (74.1)
ER neg PR neg	80 (25.9)
Molecular subtype	*n* (%)
Luminal A (LA)	233 (69.8)
Luminal B (LB)	31 (9.2)
HER-2 enriched (HER2 +)	25 (7.5)
Basal-like (BL)	45 (13.5)

ER: Estrogen receptor; PR: Progesterone receptor.

Supplementary Table 2: Basic information of the IL-12 signaling axis SNPs

Gene	Chromosome location	SNP	SNP type
IL12A	3q25.33	rs2243123	Intronic
IL12RB1	19p13.1	rs3761041	Intronic
		rs401502	Exonic
		rs404733	3^{I} UTR
		rs438421	Intronic
IL12RB2	1p31.3	rs6693065	Intronic
JAK2	9p24.1	rs10974947	Intronic
		rs2274471	Intronic
		rs7849191	Intronic
TYK2	19p13.2	rs280500	5^{1} UTR
STAT4	2q32.3	rs10168266	Intronic
		rs4274624	Intronic
		rs7599504	Intronic
		rs925847	Intronic
IFNG	12q15	rs2069718	Intronic
TBX21	17q21.32	rs2158079	Intronic
PIAS2	18q21.1	rs10502878	3^{1} UTR
		rs2156049	Intronic
		rs9304337	Intronic

p: chromosome small arm; q: chromosome large arm; UTR: Untranslated region.

Supplementary Table 3: Association of the IL-12 signaling SNPs and BC risk in Puerto Rican women by crude logistic regression analysis. See Supplementary Table 3

Supplementary Table 4: Association of IL-12 signaling SNPs with BC risk in Puerto Rican women by logistic regression. See Supplementary Table 4
