# Supplementary material for: SNPs in the interleukin-12 signaling pathway are associated with breast cancer risk in Puerto Rican women
Source: Oncotarget. 2020 Sep 15;11(37):3420–31. doi: 10.18632/oncotarget.27707 (PMC7500104; doi:10.18632/oncotarget.27707)
Supplement: Supplementary file 2 [file oncotarget-11-3420-s002.docx]

**Supplementary Table 3: Association of the IL-12 signaling SNPs and BC risk in Puerto Rican women by crude logistic regression analysis**

| **Gene** | **SNP** | **Minor**  **allele** | **Major**  **allele** | **DOM**  **p value** | **OR (95% CI)** | **ADD**  **p value** | **OR (95% CI)** | **REC**  **p value** | **OR (95% CI)** | **ALL**  **p value** | **OR (95% CI)** |
| --- | --- | --- | --- | --- | --- | --- | --- | --- | --- | --- | --- |
| *IL12A* | rs2243123 | C | T | 0.02 | 1.35 (1.04-1.75) | 0.21 | 1.14 (0.93-1.40) | 0.20 | 0.72 (0.44-1.18) | 0.21 | 1.14 (0.93-1.39) |
| *IL12A* | rs582054 | A | T | 0.29 | 1.17 (0.88-1.56) | 0.82 | 0.98 (0.81-1.18) | 0.12 | 0.77 (0.56-1.07) | 0.90 | 0.99 (0.82-1.19) |
| *IL12A* | rs17810546 | G | A | 0.24 | 1.23 (0.87-1.76) | 0.22 | 1.24 (0.88-1.75) | 0.56 | 2.06 (0.20-44.38) | 0.23 | 1.22 (0.88-1.72) |
| *IL12A* | rs485497 | G | A | 0.40 | 1.13 (0.85-1.51) | 0.63 | 1.05 (0.87-1.26) | 0.92 | 0.98 (0.72-1.35) | 0.63 | 1.05 (0.87-1.26) |
| *IL12A* | rs568408 | A | G | 0.52 | 0.90 (0.65-1.24) | 0.39 | 0.88 (0.65-1.18) | 0.32 | 0.57 (0.17-1.67) | 0.38 | 0.87 (0.65-1.18) |
| *IL12A* | rs662959 | A | G | 0.58 | 0.91 (0.66-1.26) | 0.48 | 0.90 (0.68-1.20) | 0.45 | 0.67 (0.22-1.88) | 0.48 | 0.90 (0.67-1.20) |
| *IL12B* | rs3212227 | G | T | 0.09 | 0.80 (0.61-1.03) | 0.32 | 0.85 (0.69-1.03) | 0.51 | 0.85 (0.54-1.35) | 0.12 | 0.85 (0.69-1.04) |
| *IL12B* | rs2546892 | A | G | 0.90 | 0.98 (0.71-1.35) | 0.91 | 0.98 (0.74-1.31) | 0.99 | 1.00 (0.34-2.95) | 0.91 | 1.01 (0.72-1.43) |
| *IL12B* | rs2569254 | T | C | 0.61 | 0.92 (0.69-1.25) | 0.76 | 0.96 (0.74-1.25) | 0.63 | 1.24 (0.51-3.11) | 0.75 | 0.96 (0.73-1.25) |
| *IL12B* | rs2853694 | G | T | 0.86 | 0.98 (0.75-1.27) | 0.75 | 1.03 (0.86-1.23) | 0.40 | 1.16 (0.82-1.64) | 0.73 | 1.03 (0.85-1.25) |
| *IL12B* | rs3181216 | T | A | 0.47 | 0.90 (0.69-1.18) | 0.92 | 0.99 (0.81-1.21) | 0.32 | 1.25 (0.80-1.97) | 0.92 | 1.03 (0.85-1.25) |
| *IL12B* | rs730691 | T | C | 0.64 | 0.94 (0.71-1.23) | 0.78 | 0.97 (0.81-1.17) | 0.94 | 1.01 (0.72-1.43) | 0.78 | 0.97 (0.81-1.17) |
| *IL12RB1* | \| rs401502 \| \| --- \| | G | C | 0.68 | 1.06 (0.81-1.38) | 0.18 | 1.16 (0.93-1.45) | 0.009 | 2.31 (1.26-4.43) | 0.17 | 1.16 (0.93-1.45) |
| *IL12RB1* | rs1870063 | T | C | 0.09 | 1.25 (0.96- 1.62) | 0.09 | 1.19 (0.97- 1.46) | 0.33 | 1.26 (0.79- 2.03) | 0.08 | 1.21 (0.98-1.48) |
| *IL12RB1* | rs383483 | G | A | 0.09 | 1.27 (0.97-1.68) | 0.08 | 1.17 (0.98-1.40) | 0.28 | 1.19 (0.87-1.64) | 0.06 | 1.19 (0.99-1.44) |
| *IL12RB1* | rs11086087 | G | C | 0.71 | 1.07 (0.75 -1.52) | 0.66 | 1.08 (0.77-1.51) | 0.65 | 1.51 (0.25-11.48) | 0.66 | 1.08 (0.77 -1.50) |
| *IL12RB1* | rs2045386 | A | G | 0.53 | 0.92 (0.71-1.19) | 0.76 | 0.97 (0.80-1.18) | 0.70 | 1.08 (0.72-1.61) | 0.78 | 0.97 (0.80-1.18) |
| *IL12RB1* | rs2305742 | C | A | 0.40 | 1.14 (0.83-1.56) | 0.36 | 1.14 (0.86-1.52) | 0.56 | 1.37 (0.47-4.20) | 0.36 | 1.14 (0.86-1.52) |
| *IL12RB1* | rs2305743 | A | G | 0.35 | 1.15 (0.85-1.56) | 0.24 | 1.17 (0.90-1.52) | 0.25 | 1.68 (0.70-4.27) | 0.23 | 1.18 (0.90-1.54) |
| *IL12RB1* | rs3761041 | T | C | 0.69 | 1.07 (0.77-1.49) | 0.33 | 1.16 (0.86-1.57) | 0.03 | 5.19 (1.36-33.91) | 0.32 | 1.17 (0.86-1.59) |
| *IL12RB1* | rs393548 | A | T | 0.09 | 1.28 (0.96-1.70) | 0.10 | 1.23 (0.96-1.58) | 0.55 | 1.27 (0.59-2.79) | 0.10 | 1.24 (0.96-1.59) |
| *IL12RB1* | rs404733 | T | A | 0.17 | 1.20 (0.92-1.58) | 0.03 | 1.18 (0.99-1.42) | 0.09 | 1.34 (0.96-1.88) | 0.06 | 1.20 (0.99-1.44) |
| *IL12RB1* | rs436857 | A | G | 0.13 | 1.27 (0.93-1.73) | 0.19 | 1.26 (0.96-1.67) | 0.30 | 1.72 (0.63-5.10) | 0.09 | 1.27 (0.96-1.68) |
| *IL12RB1* | rs438421 | A | G | 0.04 | 1.27 (0.93-1.73) | 0.31 | 0.81 (0.67-0.98) | 0.16 | 0.77 (0.53-1.11) | 0.03 | 0.81 (0.67-0.98) |
| *IL12RB1* | rs445521 | G | T | 0.16 | 1.21 (0.93-1.58) | 0.86 | 1.14 (0.94-1.38) | 0.55 | 1.12 (0.76-1.65) | 0.19 | 1.13 (0.94-1.37) |
| *IL12RB2* | rs10889685 | C | A | 0.91 | 0.98 (0.76 -1.28) | 0.86 | 1.02 (0.84 -1.23) | 0.57 | 1.12 (0.75-1.68) | 0.85 | 1.02 (0.84-1.23) |
| *IL12RB2* | rs12142823 | T | C | 0.79 | 0.96 (0.73-1.27) | 0.93 | 1.01 (0.79-1.29) | 0.31 | 1.52 (0.68-3.53) | 0.93 | 1.01 (0.79-1.29) |
| *IL12RB2* | rs1890741 | C | G | 0.24 | 1.17 (0.90-1.52) | 0.20 | 1.14 (0.93-1.41) | 0.42 | 1.23 (0.74-2.04) | 0.20 | 1.14 (0.93-1.41) |
| *IL12RB2* | rs2066445 | T | C | 0.28 | 1.15 (0.89-1.50) | 0.19 | 1.14 (0.93-1.40) | 0.28 | 1.30 (0.81-2.11) | 0.19 | 1.15 (0.93-1.41) |
| *IL12RB2* | rs2201584 | A | G | 0.60 | 1.07 (0.82-1.41) | 0.42 | 1.10 (0.87-1.38) | 0.29 | 1.43 (0.74-2.80) | 0.41 | 1.10 (0.87-1.39) |
| *IL12RB2* | rs3790558 | T | G | 0.48 | 0.91 (0.69-1.19) | 0.45 | 0.93 (0.77-1.12) | 0.63 | 0.91 (0.63-1.33) | 0.46 | 0.93 (0.77-1.12) |
| *IL12RB2* | rs3790568 | A | G | 0.75 | 1.05 (0.77-1.43) | 0.93 | 1.01 (0.77-1.33) | 0.52 | 0.74 (0.28-1.84) | 0.93 | 1.01 (0.77-1.34) |
| *IL12RB2* | rs3977726 | C | A | 0.12 | 1.24 (0.95-1.62) | 0.07 | 1.19 (0.99-1.43) | 0.17 | 1.29 (0.90-1.84) | 0.07 | 1.19 (0.99-1.44) |
| *IL12RB2* | rs4655541 | T | A | 0.68 | 0.95 (0.73-1.23) | 0.72 | 1.04 (0.85-1.25) | 0.17 | 1.32 (0.89-1.98) | 0.71 | 1.04 (0.85-1.26) |
| *IL12RB2* | rs56187736 | G | C | 0.17 | 0.82 (0.62-1.09) | 0.23 | 0.86 (0.68-1.10) | 0.89 | 0.95 (0.47-1.91) | 0.22 | 0.86 (0.68-1.09) |
| *IL12RB2* | rs60934365 | T | C | 0.33 | 1.15 (0.88-1.50) | 0.45 | 1.09 (0.87-1.36) | 0.77 | 0.91 (0.50-1.66) | 0.45 | 1.09 (0.87-1.35) |
| *IL12RB2* | rs6679356 | C | T | 0.08 | 1.26 (0.97-1.64) | 0.09 | 1.20 (0.97-1.48) | 0.49 | 1.20 (0.71-2.03) | 0.09 | 1.20 (0.97-1.49) |
| *IL12RB2* | \| rs6693065 \| \| --- \| | G | A | 0.04 | 0.76 (0.58-0.99) | 0.31 | 0.90 (0.75-1.09) | 0.20 | 1.17 (0.80-1.10) | 0.30 | 0.90 (0.75 -1.09) |
| *IL12RB2* | rs72678531 | C | T | 0.25 | 1.17 (0.89-1.53) | 0.42 | 1.18 (0.94-1.49) | 0.16 | 1.59 (0.84-3.08) | 0.14 | 1.19 (0.95-1.49) |
| *IL12RB2* | rs729188 | C | T | 0.15 | 0.82 (0.62-1.08) | 0.22 | 0.89 (0.74-1.07) | 0.63 | 0.92 (0.66-1.28) | 0.67 | 0.96 (0.80-1.16) |
| *IL12RB2* | rs7544381 | T | C | 0.61 | 0.93 (0.71-1.22) | 0.29 | 0.90 (0.75-1.09) | 0.17 | 0.77 (0.53-1.11) | 0.29 | 0.90 (0.75-1.09) |

| **Gene** | **SNP** | **Minor**  **allele** | **Major**  **allele** | **DOM**  **p value** | **OR (95% CI)** | **ADD**  **p value** | **OR (95% CI)** | **REC**  **p value** | **OR (95% CI)** | **ALL**  **p value** | **OR (95% CI)** |
| --- | --- | --- | --- | --- | --- | --- | --- | --- | --- | --- | --- |
| *JAK2* | rs10974947 | A | G | 0.03 | 0.74 (0.57-0.96) | 0.07 | 0.82 (0.66-1.01) | 0.82 | 0.94 (0.54-1.61) | 0.06 | 0.81 (0.66-1.01) |
| *JAK2* | rs1887428 | G | C | 0.83 | 1.03 (0.79-1.33) | 0.51 | 1.07 (0.87-1.31) | 0.26 | 1.30 (0.82-2.07) | 0.21 | 1.13 (0.93-1.38) |
| *JAK2* | rs1887429 | T | G | 0.05 | 0.76 (0.59-1.38) | 0.09 | 0.85 (0.70-1.03) | 0.20 | 0.91 (0.61-1.13) | 0.09 | 0.84 (0.69-1.02) |
| *JAK2* | rs10815148 | A | T | 0.67 | 0.94 (0.73-1.22) | 0.53 | 0.94 (0.77-1.14) | 0.51 | 0.86 (0.55-1.34) | 0.53 | 0.94 (0.77-1.14) |
| *JAK2* | rs10815162 | C | G | 0.87 | 1.02 (0.79 -1.33) | 0.96 | 0.99 (0.81-1.22) | 0.69 | 0.90 (0.55-1.47) | 0.96 | 0.99 (0.81-1.23) |
| *JAK2* | rs1536800 | T | C | 1.00 | 1.00 (0.75-1.32) | 0.85 | 1.02 (0.80-1.30) | 0.57 | 1.23 (0.60-2.56) | 0.85 | 1.02 (0.80-1.30) |
| *JAK2* | rs2230722 | T | C | 0.05 | 0.77 (0.59-1.00) | 0.14 | 0.86 (0.71-1.05) | 0.89 | 0.97 (0.65-1.45) | 0.12 | 0.86 (0.70-1.04) |
| *JAK2* | rs2230724 | G | A | 0.35 | 1.14 (0.87-1.51) | 0.93 | 0.99 (0.82-1.19) | 0.18 | 0.79 (0.57-1.11) | 0.90 | 0.99 (0.82-1.19) |
| *JAK2* | rs2274471 | G | A | 0.03 | 0.75 (0.58-0.98) | 0.02 | 0.78 (0.63-0.97) | 0.16 | 0.68 (0.40-1.15) | 0.02 | 0.78 (0.63-0.96) |
| *JAK2* | rs2274472 | C | T | 0.14 | 1.21 (0.94-1.57) | 0.22 | 1.13 (0.93-1.37) | 0.79 | 1.06 (0.69-1.61) | 0.22 | 1.13 (0.93-1.38) |
| *JAK2* | rs3780378 | T | C | 0.32 | 1.16 (0.87-1.56) | 0.58 | 1.05 (0.88-1.27) | 0.90 | 0.98 (0.72-1.33) | 0.58 | 1.05 (0.88-1.26) |
| *JAK2* | rs3780379 | A | G | 0.65 | 0.94 (0.71-1.23) | 0.64 | 0.95 (0.75-1.19) | 0.79 | 0.92 (0.47-1.76) | 0.63 | 0.94 (0.75-1.19) |
| *JAK2* | rs7030260 | A | C | 0.85 | 1.02 (0.79-1.33) | 0.66 | 1.04 (0.86-1.26) | 0.67 | 1.09 (0.72-1.65) | 0.73 | 1.03 (0.85-1.26) |
| *JAK2* | rs7046736 | A | C | 0.14 | 0.86 (0.71-1.05) | 0.70 | 0.96 (0.78-1.18) | 0.93 | 1.02 (0.66-1.57) | 0.70 | 0.96 (0.78-1.18) |
| *JAK2* | rs7849191 | T | C | 0.93 | 0.99 (0.82-1.19) | 0.55 | 1.06 (0.88-1.26) | 0.81 | 0.96 (0.71-1.30) | 0.55 | 1.06 (0.88-1.27) |
| *TYK2* | rs280500 | G | A | 0.26 | 1.17 (0.89-1.53) | 0.09 | 1.22 (0.97-1.54) | 0.04 | 2.10 (1.06-4.39) | 0.09 | 1.22 (0.97-1.54) |
| *TYK2* | rs12720270 | A | G | 0.74 | 1.05 (0.79-1.38) | 0.56 | 1.07 (0.85-1.36) | 0.36 | 1.41 (0.68-2.97) | 0.55 | 1.08 (0.84-1.37) |
| *TYK2* | rs2304256 | A | C | 0.47 | 1.10 (0.85-1.43) | 0.51 | 1.07 (0.87-1.32) | 0.82 | 1.06 (0.63-1.78) | 0.50 | 1.08 (0.87-1.33) |
| *TYK2* | rs280519 | A | G | 0.19 | 0.82 (0.61-1.11) | 0.08 | 0.85 (0.70-1.02) | 0.13 | 0.79 (0.58-1.07) | 0.09 | 0.85 (0.71-1.02) |
| *TYK2* | rs280521 | A | G | 0.85 | 0.85 (0.63-1.14) | 0.37 | 0.88 (0.67-1.16) | 0.73 | 1.21 (0.40-3.80) | 0.38 | 0.89 (0.68-1.16) |
| *STAT4* | rs925847 | T | C | 0.08 | 0.79 (0.61-1.36) | 0.04 | 0.81 (0.67-0.99) | 0.22 | 0.70 (0.46-1.08) | 0.04 | 0.81 (0.66-0.99) |
| *STAT4* | rs3821236 | A | G | 0.08 | 0.79 (0.61-1.03) | 0.07 | 0.83 (0.68-1.02) | 0.31 | 0.79 (0.50-1.24) | 0.07 | 0.83 (0.68-1.02) |
| *STAT4* | rs10168266 | T | C | 0.03 | 0.75 (0.57-0.97) | 0.10 | 0.84 (0.69-1.03) | 0.87 | 1.04 (0.65-1.67) | 0.09 | 0.84 (0.68-1.03) |
| *STAT4* | rs10176621 | C | T | 0.52 | 1.09 (0.84-1.41) | 0.33 | 1.10 (0.91-1.34) | 0.29 | 1.26 (0.82-1.93) | 0.32 | 1.10 (0.91-1.35) |
| *STAT4* | rs10181819 | C | T | 0.13 | 0.81 (0.62-1.07) | 0.34 | 0.90 (0.72-1.12) | 0.57 | 1.17 (0.67-2.06 | 0.31 | 0.89 (0.71-1.11) |
| *STAT4* | rs1031508 | A | G | 0.23 | 0.85 (0.66-1.10) | 0.22 | 0.89 (0.73-1.07) | 0.61 | 0.90 (0.60-1.35) | 0.25 | 0.89 (0.73-1.08) |
| *STAT4* | rs12463658 | A | C | 0.62 | 0.93 (0.71-1.23) | 0.65 | 1.04 (0.87-1.26) | 0.12 | 1.31 (0.93-1.84) | 0.60 | 1.05 (0.87-1.26) |
| *STAT4* | rs1517352 | C | A | 0.40 | 0.89 (0.67-1.17) | 0.77 | 1.03 (0.85-1.24) | 0.11 | 1.31 (0.94-1.84) | 0.73 | 1.03 (0.86-1.24) |
| *STAT4* | rs16833220 | G | C | 0.10 | 0.77 (0.56-1.05) | 0.29 | 0.86 (0.65-1.14) | 0.11 | 2.61 (0.87-9.57) | 0.29 | 0.86 (0.65-1.14) |
| *STAT4* | rs16833260 | C | G | 0.19 | 0.83 (0.64-1.09) | 0.34 | 0.91 (0.76-1.10) | 0.95 | 0.99 (0.69-1.42) | 0.33 | 0.91 (0.75-1.10) |
| *STAT4* | rs2356350 | G | A | 0.79 | 1.04 (0.79-1.35) | 0.72 | 1.03 (0.86-1.25) | 0.75 | 1.06 (0.73-1.54) | 0.72 | 1.03 (0.86-1.25) |
| *STAT4* | rs3024866 | G | A | 0.57 | 0.92 (0.69-1.22) | 0.95 | 0.99 (0.83-1.20) | 0.59 | 1.09 (0.79-1.51) | 0.95 | 0.99 (0.83-1.19) |
| *STAT4* | rs3024879 | A | G | 0.51 | 1.11 (0.81-1.53) | 0.24 | 1.18 (0.89-1.57) | 0.05 | 3.12 (1.08-11.24) | 0.10 | 1.27 (0.95-1.69) |
| *STAT4* | rs3024896 | T | C | 0.07 | 0.77 (0.58-1.02) | 0.08 | 0.81 (0.64-1.02) | 0.51 | 0.80 (0.40-1.56) | 0.07 | 0.80 (0.63-1.02) |
| *STAT4* | rs3024897 | C | G | 0.42 | 0.87(0.63-1.21) | 0.54 | 0.90 (0.66-1.24) | 0.34 | 3.03 (0.38-61.31) | 0.55 | 0.91 (0.67-1.24) |
| *STAT4* | rs34675442 | A | G | 0.61 | 0.92 (0.65-1.29) | 0.83 | 0.96 (0.70-1.33) | 0.27 | 2.53 (0.54-17.71) | 0.83 | 0.96 (0.70-1.33) |
| *STAT4* | rs4274624 | C | T | 0.02 | 0.74 (0.57-0.96) | 0.07 | 0.83 (0.68-1.01) | 0.77 | 0.93 (0.60-1.46) | 0.06 | 0.82 (0.67-1.01) |
| *STAT4* | rs4853540 | T | G | 0.62 | 0.94 (0.72-1.21) | 0.69 | 0.96 (0.79-1.17) | 0.95 | 0.99 (0.64-1.53) | 0.69 | 0.96 (0.79-1.17) |
| *STAT4* | rs4853543 | A | G | 0.13 | 0.81 (0.62-1.06) | 0.34 | 0.91 (0.76-1.10) | 0.86 | 1.03 (0.73-1.46) | 0.33 | 0.91 (0.75-1.10) |
| *STAT4* | rs6434435 | A | G | 0.73 | 0.95 (0.73-1.25) | 0.82 | 0.97 (0.78-1.22) | 0.85 | 1.06 (0.56-2.03) | 0.82 | 0.97 (0.77-1.22) |
| *STAT4* | rs6738544 | A | C | 0.28 | 0.86 (0.66-1.13) | 0.74 | 0.97 (0.80-1.17) | 0.43 | 1.16 (0.81-1.66) | 0.74 | 0.97 (0.80-1.17) |

| **Gene** | **SNP** | **Minor**  **allele** | **Major**  **allele** | **DOM**  **p value** | **OR (95% CI)** | **ADD**  **p value** | **OR (95% CI)** | **REC**  **p value** | **OR (95% CI)** | **ALL**  **p value** | **OR (95% CI)** |
| --- | --- | --- | --- | --- | --- | --- | --- | --- | --- | --- | --- |
| *STAT4* | rs72913168 | T | C | 0.60 | 0.91 (0.641.28) | 0.75 | 0.95 (0.69-1.31) | 0.45 | 1.74 (0.42-8.52) | 0.75 | 0.95 (0.69-1.31) |
| *STAT4* | rs7572482 | A | G | 0.61 | 0.93 (0.71-1.22) | 0.69 | 0.97 (0.81-1.17) | 0.89 | 1.02 (0.73-1.44) | 0.78 | 0.97 (0.81-1.17) |
| *STAT4* | rs7595886 | T | C | 0.84 | 1.03 (0.77-1.36) | 0.80 | 1.03 (0.81-1.31) | 0.81 | 1.09 (0.52-2.32) | 0.80 | 1.03 (0.81-1.32) |
| *STAT4* | rs7599504 | C | T | 0.74 | 0.95 (0.68-1.31) | 0.76 | 1.04 (0.78-1.40) | 0.04 | 3.85 (1.19-17.12) | 0.76 | 1.05 (0.78-1.40) |
| *STAT4* | rs3024847 | A | T | 0.51 | 0.91 (0.69-1.20) | 0.71 | 1.03 (0.86-1.25) | 0.13 | 1.30 (0.92-1.85) | 0.71 | 1.03 (0.86-1.25) |
| *STAT4* | rs6752770 | G | A | 0.39 | 0.89 (0.69-1.16) | 0.23 | 0.88 (0.72-1.08) | 0.22 | 0.76 (0.48-1.18) | 0.22 | 0.88 (0.72-1.08) |
| *IFNG* | rs1861494 | C | T | 0.65 | 1.06 (0.82-1.38) | 0.66 | 1.05 (0.84-1.31) | 0.87 | 1.05 (0.58-1.93) | 0.66 | 1.05 (0.84-1.30) |
| *IFNG* | rs2069705 | G | A | 0.50 | 1.10 (0.83-1.44) | 0.30 | 1.10 (0.91-1.34) | 0.29 | 1.21 (0.85-1.74) | 0.31 | 1.10 (0.91-1.33) |
| *IFNG* | rs2069718 | G | A | 0.12 | 0.79 (0.59-1.06) | 0.04 | 0.82 (0.68-0.99) | 0.08 | 0.75 (0.55-1.03) | 0.05 | 0.83 (0.69-1.00) |
| *IFNG* | rs2069727 | C | T | 0.94 | 0.99 (0.76-1.28) | 0.54 | 0.94 (0.78-1.14) | 0.22 | 0.77 (0.51-1.16) | 0.51 | 0.94 (0.77-1.14) |
| *TBX21* | rs11650354 | T | C | 0.54 | 0.91 (0.68-1.22) | 0.27 | 0.87 (0.67- 1.11) | 0.06 | 0.43 (0.16- 1.02) | 0.27 | 0.87 (0.68-1.11) |
| *TBX21* | rs11652969 | A | G | 0.75 | 1.04 (0.80-1.37) | 0.87 | 1.01 (0.84-1.22) | 0.86 | 0.97 (0.68-1.37) | 0.90 | 1.01 (0.84-1.22) |
| *TBX21* | rs11657388 | G | C | 0.50 | 1.09 (0.84-1.42) | 0.94 | 1.01 (0.82-1.24) | 0.31 | 0.76 (0.44-1.28) | 0.89 | 1.01 (0.82-1.25) |
| *TBX21* | rs16946264 | A | G | 0.22 | 1.22 (0.89-1.67) | 0.30 | 1.16 (0.87-1.55) | 0.79 | 0.86 (0.28-2.62) | 0.30 | 1.16 (0.87-1.56) |
| *TBX21* | rs2158079 | C | T | 0.94 | 0.99 (0.74-1.33) | 0.52 | 1.08 (0.85-1.39) | 0.04 | 2.30 (1.06-5.36) | 0.50 | 1.09 (0.84-1.42) |
| *SOCS3* | rs4969169 | T | C | 0.80 | 0.95 (0.66-1.37) | 0.59 | 0.91 (0.66-1.26) | 0.26 | 0.50 (0.13-1.59) | 0.52 | 0.90 (0.64-1.25) |
| *SOCS3* | rs4969170 | A | G | 0.89 | 1.02 (0.77-1.36) | 0.94 | 0.99 (0.83-1.19) | 0.78 | 0.95 (0.69-1.31) | 0.94 | 0.99 (0.83-1.19) |
| *PIAS2* | rs10502878 | T | C | 0.03 | 0.73 (0.55-1.25) | 0.05 | 0.77 (0.59-1.00) | 0.83 | 0.90 (0.33-2.38) | 0.05 | 0.77 (0.60-1.00) |
| *PIAS2* | rs10853545 | A | T | 0.60 | 0.92 (0.69-1.24) | 0.24 | 0.90 (0.75-1.07) | 0.22 | 1.09 (0.79-1.53) | 0.28 | 0.90 (0.75-1.08) |
| *PIAS2* | rs2156049 | A | G | 0.07 | 1.28 (0.98-1.68) | 0.13 | 1.15 (0.96-1.37) | 0.59 | 1.09 (0.79-1.53) | 0.12 | 1.16 (0.96-1.39) |
| *PIAS2* | rs9304337 | G | A | 0.20 | 0.83 (0.63-1.10) | 0.02 | 0.81 (0.67-0.98) | 0.20 | 1.20 (0.91-1.57) | 0.02 | 0.81 (0.67-0.97) |

| DOM: genetic dominant model; ADD: genetic additive model; REC: Recessive genetic model; OR: Odds Ratio; 95% CI: 95% interval confidence |  |  |  |
| --- | --- | --- | --- |
