# Supplementary material for: SNPs in the interleukin-12 signaling pathway are associated with breast cancer risk in Puerto Rican women
Source: Oncotarget. 2020 Sep 15;11(37):3420–31. doi: 10.18632/oncotarget.27707 (PMC7500104; doi:10.18632/oncotarget.27707)
Supplement: Supplementary file 3 [file oncotarget-11-3420-s003.docx]

**Supplementary Table 4: Association of IL-12 signaling SNPs with BC risk in Puerto Rican women by logistic regression**

| **Gene** | **SNP** | **Model** | **Genotype** | **Cases** | **Controls** | **OR (95% CI); p value** | **OR (95% CI); p value** | **OR (95% CI); p value** |
| --- | --- | --- | --- | --- | --- | --- | --- | --- |
|  |  |  |  | **% (n)** | **% (n)** | **Crude** | **Adjusted^1^** | **Adjusted^2^** |
| *IL12A* | rs2243123 | Allelic | T | 70 (640) | 73 (674) | Ref. | Ref. | Ref. |
|  |  |  | C | 30 (268) | 27 (248) | 1.14 (0.93-1.39); 021 | 1.10 (0.89-1.37); 0.36 | 1.07 (0.84-1.36); 0.56 |
|  |  | Dominant | TT | 48 (216) | 55 (254) | Ref. | Ref. | Ref. |
|  |  |  | CT + CC | 52 (246) | 45 (207) | 1.35 (1.04-1.75); **0.02** | 1.34 (1.02-1.77); **0.04** | 1.29 (0.95-1.75); 0.10 |
|  |  | Recessive | CT+ TT | 93 (424) | 91 (424) | Ref. | Ref. | Ref. |
|  |  |  | CC | 7 (30) | 9 (41) | 0.72 (0.44-1.18); 0.20 | 0.61 (0.35-1.04); 0.08 | 0.61 (0.33-1.08); 0.10 |
|  |  | Additive | — | — | — | 1.14 (0.93-1.40); 0.21 | 1.11 (0.89-1.38); 0.36 | 1.07 (0.84-1.37); 0.56 |
| *IL12RB1* | rs3761041 | Allelic | C | 89 (805) | 91 (834) | Ref. | Ref. | Ref. |
|  |  |  | T | 11 (97) | 9 (86) | 1.17 (0.86-1.59); 0.32 | 1.14 (0.83-1.58); 0.42 | 1.19 (0.83-1.70); 0.34 |
|  |  | Dominant | CC | 81 (364) | 82 (376) | Ref. | Ref. | Ref. |
|  |  |  | TC + TT | 19 (87) | 18 (84) | 1.07 (0.77-1.49); 0.69 | 1.03 (0.72-1.46); 0.88 | 1.09 (0.74-1.61); 0.65 |
|  |  | Recessive | TC + CC | 98 (441) | 99 (458) | Ref. | Ref. | Ref. |
|  |  |  | TT | 2 (10) | 1 (2) | 5.19 (1.36-33.91); **0.03** | 6.13 (1.52-40.95); **0.02** | 4.80 (1.10-33.14); 0.06 |
|  |  | Additive | — | — | — | 1.16 (0.86-1.57); 0.33 | 1.14 (0.83-1.57); 0.33 | 1.18 (0.83-1.69); 0.35 |
|  | rs401502 | Allelic | C | 79 (726) | 76 (697) | Ref. | Ref. | Ref. |
|  |  |  | G | 21 (194) | 24 (217) | 1.16 (0.93-1.45); 0.17 | 1.18 (0.93-1.49); 0.17 | 1.13 (0.87-1.46); 0.35 |
|  |  | Dominant | CC | 61 (281) | 60 (273) | Ref. | Ref. | Ref. |
|  |  |  | CG + GG | 39 (179) | 40 (184) | 1.06 (0.81-1.38); 0.68 | 1.05 (0.79-1.39); 0.72 | 0.99 (0.73-1.35); 0.97 |
|  |  | Recessive | CG + CC | 97 (445) | 93 (424) | Ref. | Ref. | Ref. |
|  |  |  | GG | 3 (15) | 7 (33) | 2.31 (1.26-4.43); **0.009** | 2.65 (1.37-5.36); **0.005** | 2.68 (1.29-5.68); **0.009** |
|  |  | Additive | — | — | — | 1.16 (0.93-1.45); 0.18 | 1.18 (0.93-1.49); 0.18 | 1.13 (0.87-1.47); 0.35 |
|  | rs404733 | Allelic | A | 57 (518) | 61 (565) | Ref. | Ref. | Ref. |
|  |  |  | T | 43 (394) | 39 (359) | 1.20 (0.99-1.44); 0.06 | 1.26 (1.03-1.54); **0.02** | 1.20 (0.96-1.49); 0.11 |
|  |  | Dominant | AA | 34 (156) | 39 (178) | Ref. | Ref. | Ref. |
|  |  |  | AT + TT | 66 (300) | 61 (284) | 1.20 (0.92-1.58); 0.17 | 1.29 (0.97-1.72); 0.08 | 1.23 (0.90-1.70); 0.19 |
|  |  | Recessive | AT+ AA | 79 (362) | 83 (387) | Ref. | Ref. | Ref. |
|  |  |  | TT | 21 (94) | 17 (75) | 1.34 (0.96-1.88); 0.09 | 1.43 (1.00-2.05); 0.05 | 1.29 (0.87-1.92); 0.20 |
|  |  | Additive | — | — | — | 1.18 (0.99-1.42); 0.07 | 1.24 (1.02-1.51); **0.03** | 1.18 (0.96-1.47); 0.12 |
|  | rs438421 | Allelic | G | 63 (574) | 58 (536) | Ref. | Ref. | Ref. |
|  |  |  | A | 37 (332) | 42 (382) | 0.81 (0.67-0.98); **0.03** | 0.80 (0.65-0.98); **0.03** | 0.78 (0.63-0.97); **0.03** |
|  |  | Dominant | GG | 40 (182) | 34 (154) | Ref. | Ref. | Ref. |
|  |  |  | GA + GG | 60 (271) | 66 (305) | 0.75 (0.57-0.98); **0.04** | 0.75 (0.56-0.99); 0.05 | 0.71 (0.52-0.97); **0.03** |
|  |  | Recessive | GA + GG | 86 (392) | 83 (382) | Ref. | Ref. | Ref. |
|  |  |  | AA | 14 (61) | 17 (77) | 0.77 (0.53-1.11); 0.16 | 0.74 (0.50-1.09); 0.13 | 0.75 (0.48-1.14); 0.18 |
|  |  | Additive | — | — | — | 0.81 (0.67-0.98); **0.03** | 0.80 (0.65-0.98); **0.03** | 0.78 (0.62-0.97); **0.03** |

.

| **Gene** | **SNP** | **Model** | **Genotype** | **Cases** | **Controls** | **OR (95% CI); p value** | **OR (95% CI); p value** | **OR (95% CI); p value** |
| --- | --- | --- | --- | --- | --- | --- | --- | --- |
|  |  |  |  | **% (n)** | **% (n)** | **Crude** | **Adjusted** | **Adjusted** |
| *IL12RB2* | rs6693065 | Allelic | A | 64 (572) | 62 (566) | Ref. | Ref. | Ref. |
|  |  |  | G | 36 (320) | 38 (350) | 0.90 (0.75-1.09); 0.30 | 0.93 (0.76-1.15); 0.52 | 0.85 (0.68-1.07); 0.17 |
|  |  | Dominant | AA | 43 (193) | 47 (216) | Ref. | Ref. | Ref. |
|  |  |  | AG + GG | 57 (253) | 53 (290) | 0.76 (0.58-0.99); **0.04** | 0.82 (0.61-1.09); 0.14 | 0.82 (0.61-1.30); 0.17 |
|  |  | Recessive | AG + AA | 85 (379) | 87 (398) | Ref. | Ref. | Ref. |
|  |  |  | GG | 15 (67) | 13 (60) | 1.17 (0.80-1.10); 0.20 | 1.15 (0.77-1.72); 0.49 | 0.92 (0.58-1.46); 0.72 |
|  |  | Additive | — | — | — | 0.90 (0.75-1.09); 0.31 | 0.93 (0.76-1.15); 0.52 | 0.85 (0.68-1.07). 0.17 |
| *JAK2* | rs10974947 | Allelic | G | 78 (697) | 74 (671) | Ref. | Ref. | Ref. |
|  |  |  | A | 22 (199) | 26 (235) | 0.81 (0.66-1.01); 0.06 | 0.83 (0.66-1.04); 0.11 | 0.83 (0.64-1.06); 0.14 |
|  |  | Dominant | GG | 62 (277) | 55 (247) | Ref. | Ref. | Ref. |
|  |  |  | GA + AA | 38 (171) | 45 (206) | 0.74 (0.57-0.96); **0.03** | 0.73 (0.55-0.97); **0.01** | 0.77 (0.56-1.05); **0.04** |
|  |  | Recessive | GA + GG | 94 (420) | 94 (424) | Ref. | Ref. | Ref. |
|  |  |  | AA | 6 (28) | 6 (29) | 0.94 (0.54-1.61); 0.82 | 1.06 (0.60-1.89); 0.83 | 0.85 (0.44-1.62); 0.62 |
|  |  | Additive | — | — | — | 0.82 (0.66-1.01); 0.07 | 0.83 (0.66-1.04); 0.11 | 0.83 (0.64-1.06); 0.14 |
|  | rs2274471 | Allelic | A | 78 (682) | 73 (667) | Ref. | Ref. | Ref. |
|  |  |  | G | 22 (198) | 27 (249) | 0.78 (0.63-0.96); **0.02** | 0.74 (0.59-0.93); **0.01** | 0.70 (0.54-0.90); **0.006** |
|  |  | Dominant | AA | 61(267) | 54 (246) | Ref. | Ref. | Ref. |
|  |  |  | CG + GG | 39 (173) | 46 (212) | 0.75 (0.58-0.98); **0.03** | 0.74 (0.56-0.99); **0.04** | 0.72 (0.53-0.99); **0.04** |
|  |  | Recessive | AG + AA | 94(415) | 92 (421) | Ref. | Ref. | Ref. |
|  |  |  | GG | 6 (25) | 8 (37) | 0.68 (0.40-1.15); 0.16 | 0.52 (0.28-0.93); **0.03** | 0.39 (0.19-0.76); **0.007** |
|  |  | Additive | — | — | — | 0.78 (0.63-0.97); **0.02** | 0.75 (0.59-0.94); **0.01** | 0.70 (0.54-0.91); **0.007** |
|  | rs7849191 | Allelic | C | 50 (453) | 51 (473) | Ref. | Ref. | Ref. |
|  |  |  | T | 50 (455) | 49 (449) | 1.06 (0.88-1.27); 0.55 | 1.06 (0.87-1.28); 0.58 | 1.11 (0.90-1.38); 0.33 |
|  |  | Dominant | CC | 25 (112) | 28 (130) | Ref. | Ref. | Ref. |
|  |  |  | CT + TT | 75 (342) | 72 (331) | 1.20 (0.89-1.61); 0.23 | 1.25 (0.91-1.72); 0.16 | 1.49 (1.05-2.13); **0.02** |
|  |  | Recessive | CT + CC | 75 (341) | 74 (343) | Ref. | Ref. | Ref. |
|  |  |  | TT | 25 (113) | 26 (118) | 0.96 (0.71-1.30); 0.81 | 0.92 (0.67-1.27); 0.61 | 0.89 (0.62-1.26); 0.50 |
|  |  | Additive | — | — | — | 1.06 (0.88-1.26); 0.55 | 1.05 (0.87-1.28); 0.58 | 1.11 (0.90-1.37); 0.33 |
| *TYK2* | rs280500 | Allelic | A | 79 (712) | 82 (755) | Ref. | Ref. | Ref. |
|  |  |  | G | 21 (190) | 18 (165) | 1.22 (0.97-1.54); 0.09 | 1.25 (0.98-1.60); 0.07 | 1.36 (1.03-1.78); **0.03** |
|  |  | Dominant | AA | 63 (285) | 67 (307) | Ref. | Ref. | Ref. |
|  |  |  | GA + GG | 37 (166) | 37 (153) | 1.17 (0.89-1.53); 0.26 | 1.19 (0.89-1.03); 0.18 | 1.27 (0.92-1.76); 0.14 |
|  |  | Recessive | GA + AA | 95 (427) | 98 (448) | Ref. | Ref. | Ref. |
|  |  |  | GG | 5 (24) | 2 (12) | 2.10 (1.06-4.39); **0.04** | 2.25 (1.09-4.88); **0.03** | 2.86 (1.31-6.64); **0.01** |
|  |  | Additive | — | — | — | 1.22 (0.97-1.54); 0.09 | 1.25 (0.98-1.60); 0.08 | 1.35 (1.03-1.77); **0.02** |

| **Gene** | **SNP** | **Model** | **Genotype** | **Cases** | **Controls** | **OR (95% CI); p value** | **OR (95% CI); p value** | **OR (95% CI); p value** |
| --- | --- | --- | --- | --- | --- | --- | --- | --- |
|  |  |  |  | **% (n)** | **% (n)** | **Crude** | **Adjusted** | **Adjusted** |
| *STAT4* | rs10168266 | Allelic | C | 75 (683) | 71 (657) | Ref. | Ref. | Ref. |
|  |  |  | T | 25 (231) | 29 (265) | 0.84 (0.68-1.03); 0.09 | 0.80 (0.64-1.00); 0.05 | 0.81 (0.64-1.04); 0.09 |
|  |  | Dominant | CC | 58 (264) | 51 (233) | Ref. | Ref. | Ref. |
|  |  |  | CT + TT | 42 (193) | 49 (228) | 0.75 (0.57-0.97); **0.03** | 0.71 (0.54-0.94); **0.02** | 0.72 (0.53-0.98); **0.04** |
|  |  | Recessive | CT + CC | 92 (419) | 92 (424) | Ref. | Ref. | Ref. |
|  |  |  | TT | 8 (38) | 8 (37) | 1.04 (0.65-1.67); 0.87 | 0.97 (0.58-1.61); 0.91 | 0.98 (0.56-1.72); 0.95 |
|  |  | Additive | — | — | — | 0.84 (0.69-1.03); 0.10 | 0.81 (0.65-1.00); 0.06 | 0.82 (0.64-1.04); 0.10 |
|  | rs4274624 | Allelic | T | 74 (672) | 69 (641) | Ref. | Ref. | Ref. |
|  |  |  | C | 26 (242) | 31 (281) | 0.82 (0.67-1.01); 0.06 | 0.80 (0.64-0.99); **0.04** | 0.78 (0.61-0.99); **0.04** |
|  |  | Dominant | TT | 56 (257) | 49 (225) | Ref. | Ref. | Ref. |
|  |  |  | CT + CC | 44 (200) | 51 (236) | 0.74 (0.57-0.96); **0.02** | 0.73 (0.55-0.96); **0.02** | 0.94 (0.65-1.37); **0.04** |
|  |  | Recessive | CT + TT | 91 (415) | 90 (416) | Ref. | Ref. | Ref. |
|  |  |  | CC | 9 (42) | 10 (45) | 0.93 (0.60-1.46); 0.77 | 0.85 (0.52-1.36); 0.50 | 0.75 (0.44-1.29); 0.31 |
|  |  | Additive | — | — | — | 0.83 (0.68-1.01); 0.07 | 0.81 (0.65-0.99); 0.05 | 0.78 (0.62-0.99); **0.04** |
|  | rs7599504 | Allelic | T | 89 (783) | 89 (812) | Ref. | Ref. | Ref. |
|  |  |  | C | 11 (101) | 11 (100) | 1.05 (0.78-1.40); 0.76 | 0.98 (0.71-1.34); 0.98 | 1.00 (0.70-1.42); 0.99 |
|  |  | Dominant | TT | 80 (352) | 79 (359) | Ref. | Ref. | Ref. |
|  |  |  | CT + CC | 20 (90) | 21 (97) | 0.95 (0.68-1.31); 0.74 | 0.87 (0.62-1.24); 0.45 | 0.89 (0.61-1.31); 0.56 |
|  |  | Recessive | CT + TT | 98 (431) | 99 (453) | Ref. | Ref. | Ref. |
|  |  |  | CC | 2 (11) | 1 (3) | 3.85 (1.19-17.12); **0.04** | 3.80 (1.11-17.54); 0.05 | 4.74 (1.08-33.36); 0.06 |
|  |  | Additive | — | — | — | 1.04 (0.78-1.40); 0.76 | 0.98 (0.72-1.34); 0.91 | 1.00 (0.71-1.42); 0.99 |
|  | rs925847 | Allelic | C | 70 (642) | 66 (603) | Ref. | Ref. | Ref. |
|  |  |  | T | 30 (270) | 34 (313) | 0.84 (0.68-1.030; 0.09 | 0.83 (0.67-1.02); 0.08 | 0.85 (0.76-1.07); 0.17 |
|  |  | Dominant | CC | 49 (226) | 44 (201) | Ref. | Ref. | Ref. |
|  |  |  | TC + TT | 51 (230) | 56 (257) | 0.79 (0.61-1.36); 0.08 | 0.82 (0.62-1.09); 0.14 | 0.82 (0.62-1.09); 0.17 |
|  |  | Recessive | TC + CC | 91 (416) | 88 (403) | Ref. | Ref. | Ref. |
|  |  |  | TT | 9 (40) | 12 (55) | 0.70 (0.46-1.08); 0.22 | 0.72 (0.45-1.15); 0.17 | 0.79 (0.47-1.33); 0.39 |
|  |  | Additive | — | — | — | 0.81 (0.67-0.99); **0.04** | 0.84 (0.68-1.03); 0.10 | 0.85 (0.67-1.08); 0.19 |
| *IFNG* | rs2069718 | Allelic | A | 55 (497) | 51 (465) | Ref. | Ref. | Ref. |
|  |  |  | G | 45 (405) | 49 (457) | 0.83 (0.69-1.00); 0.05 | 0.75 (0.61-0.91); **0.004** | 0.73 (0.58-0.90); **0.004** |
|  |  | Dominant | AA | 29 (133) | 25 (113) | Ref. | Ref. | Ref. |
|  |  |  | AG + GG | 71 (318) | 75 (346) | 0.79 (0.59-1.06); 0.12 | 0.68 (0.50-0.93); **0.02** | 0.63 (0.44-0.88); **0.007** |
|  |  | Recessive | AG + AA | 81 (364) | 76 (350) | Ref. | Ref. | Ref. |
|  |  |  | GG | 19 (87) | 24 (111) | 0.75 (0.55-1.03); 0.08 | 0.66 (0.47-0.93); **0.02** | 0.68 (0.47-0.99); 0.05 |
|  |  | Additive | — | — | — | 0.82 (0.68-0.99); **0.04** | 0.74 (0.60-0.91); **0.004** | 0.72 (0.58-0.90); **0.004** |

| **Gene** | **SNP** | **Model** | **Genotype** | **Cases** | **Controls** | **OR (95% CI); p value** | **OR (95% CI); p value** | **OR (95% CI); p value** |
| --- | --- | --- | --- | --- | --- | --- | --- | --- |
|  |  |  |  | **% (n)** | **% (n)** | **Crude** | **Adjusted** | **Adjusted** |
| *TBX21* | rs2158079 | Allelic | T | 85 (778) | 86 (795) | Ref. | Ref. | Ref. |
|  |  |  | C | 15 (138) | 14 (129) | 1.09 (0.84-1.42); 0.50 | 1.08 (0.82-1.42); 0.58 | 1.19 (0.88-1.61); 0.24 |
|  |  | Dominant | TT | 74 (340) | 74 (342) | Ref. | Ref. | Ref. |
|  |  |  | CT + CC | 26 (118) | 26 (120) | 0.99 (0.74-1.33); 0.94 | 0.98 (0.72-1.35); 0.92 | 1.07 (0.76-1.51); 0.68 |
|  |  | Recessive | CT + TT | 96 (438) | 98 (453) | Ref. | Ref. | Ref. |
|  |  |  | CC | 4 (20) | 2 (9) | 2.30 (1.06-3.56); **0.04** | 2.24 (0.97-5.63); 0.07 | 2.86 (1.31-6.64); **0.02** |
|  |  | Additive | — | — | — | 1.08 (0.85-1.39); 0.52 | 1.07 (0.76-1.51); 0.59 | 1.18 (0.88-1.58); 0.26 |
| *PIAS2* | rs10502878 | Allelic | C | 83 (768) | 87 (786) | Ref. | Ref. | Ref. |
|  |  |  | T | 14 (129) | 13 (122) | 0.77 (0.60-1.00); 0.05 | 0.78 (0.59-1.02); 0.08 | 0.81 (0.60-1.090; 0.18 |
|  |  | Dominant | CC | 68 (316) | 75 (340) | Ref. | Ref. | Ref. |
|  |  |  | TC + TT | 32 (145) | 25 (114) | 0.73 (0.55-1.25); **0.03** | 0.75 (0.55-1.03); 0.16 | 0.78 (0.56-1.04); 0.15 |
|  |  | Recessive | TC + CC | 98 (452) | 98 (446) | Ref. | Ref. | Ref. |
|  |  |  | TT | 2 (9) | 2 (8) | 0.90 (0.33-2.38); 0.83 | 0.73 (0.23-2.10); 0.56 | 0.77 (0.24-2.26); 0.64 |
|  |  | Additive | — | — | — | 0.77 (0.59-1.00); 0.05 | 0.77 (0.58-1.02); 0.07 | 0.80 (0.59-1.09); 0.16 |
|  | rs2156049 | Allelic | G | 57 (521) | 60 (556) | Ref. | Ref. | Ref. |
|  |  |  | A | 43 (395) | 40 (364) | 1.16 (0.96-1.39); 0.12 | 1.22 (1.00-1.48); 0.05 | 1.20 (0.96-1.49); 0.10 |
|  |  | Dominant | GG | 33 (152) | 39 (179) | Ref. | Ref. | Ref. |
|  |  |  | GA + AA | 67 (306) | 61 (281) | 1.28 (0.98-1.68); 0.07 | 1.38 (1.03-1.84); **0.03** | 1.36 (0.98-1.87); 0.06 |
|  |  | Recessive | GA + GG | 81 (369) | 82 (377) | Ref. | Ref. | Ref. |
|  |  |  | AA | 19 (89) | 18 (83) | 1.09 (0.79-1.53); 0.59 | 1.16 (0.81-1.64); 0.41 | 1.13 (0.77-1.66); 0.54 |
|  |  | Additive | — | — | — | 1.15 (0.96-1.37); 0.13 | 1.20 (0.99-1.46); 0.06 | 1.18 (0.96-1.46); 0.11 |
|  | rs9304337 | Allelic | A | 61 (551) | 56 (657) | Ref. | Ref. | Ref. |
|  |  |  | G | 39 (347) | 44 (265) | 0.81 (0.66-0.99); **0.02** | 0.82 (0.67-1.00); 0.05 | 0.79 (0.64-0.99); **0.04** |
|  |  | Dominant | AA | 37 (166) | 33 (151) | Ref. | Ref. | Ref. |
|  |  |  | AG + GG | 63 (283) | 67 (184) | 0.83 (0.63-1.10); 0.20 | 0.88 (0.66-1.17); 0.38 | 0.85 (0.61-1.17); 0.32 |
|  |  | Recessive | AG + AA | 86 (385) | 80 (365) | Ref. | Ref. | Ref. |
|  |  |  | GG | 14 (64) | 20 (94) | 1.20 (0.91-1.57); 0.20 | 1.14 (0.85-1.52); 0.38 | 1.18 (0.85-1.62); 0.32 |
|  |  | Additive | — | — | — | 0.81 (0.67-0.99); **0.02** | 0.82 (0.67-1.00); 0.05 | 0.79 (0.64-0.99); **0.04** |

OR: Odds ratio; CI: confidence interval; ER: Estrogen receptor; PR: progesterone receptor; neg: negative

^1^Adjusted for Age and Educational level; ^2^Adjusted for Age, Educational level, ERT and Breastfeeding duration.
